# An examination of primary health care nursing service evaluation using the Donabedian model: A systematic review

**DOI:** 10.1002/nur.22291

**Published:** 2022-12-25

**Authors:** Kylie McCullough, Lesley Andrew, Angela Genoni, Melissa Dunham, Lisa Whitehead, Davina Porock

**Affiliations:** ^1^ School of Nursing and Midwifery Edith Cowan University Joondalup Western Australia Australia; ^2^ School of Medical and Health Sciences Edith Cowan University Joondalup Western Australia Australia; ^3^ Centre for Research in Aged Care Edith Cowan University Joondalup Western Australia Australia

**Keywords:** Donabedian model, evaluation, nursing, primary health care, systematic review

## Abstract

Nurses are key to the delivery of global primary health care services. However, there appears to be a lack of evaluation of primary health care nursing delivery models in the published literature. This evaluation is vital to the improvement of patient experiences, national and global health outcomes, and the justification of future investment in primary health care nursing services. The purpose of this review was to explore and analyze the literature that reports on the evaluation of primary health care nursing services, to ascertain the nature and utility of these evaluation methods, and identify opportunities for future research in this area. A systematic review of the published literature was conducted following PRISMA guidelines, using the databases CINAHL, Joanna Briggs Institute, MEDLINE, and Proquest. Thirty‐two articles published between 2010 and 2022 were selected. Results were organized using the Donabedian model. A paucity of research into the evaluation of nurse‐led primary health care services was noted. Where evident, evaluation of primary health care nursing services tended to reflect the medical model. Medical outcomes measures dominated evaluation criteria including diagnosis rates, prescription costs, and disease outcomes. Primary health care principles such as service accessibility, cultural appropriateness, and availability were rarely used. The perspectives and experiences of nurses were not sought in service evaluation, including most of the nurse‐led services. Development of an evidence‐base of nursing primary health care services that are informed by the nursing experience and apply a framework of universal primary health care principles across the structure, process, and outcomes aspects of the service is recommended.

## 1 INTRODUCTION

### 1.1 Primary care and primary health care (PHC)

The concepts of primary care and PHC are often used interchangeably by practitioners, politicians, and scholars (Keleher, 2001). In essence, primary care is a component of PHC. Defined as the first level of contact for individuals seeking help in the community (usually the general practitioner setting), primary care is based on the biomedical model of health and usually involves a “one‐off” intervention where the patient seeks a treatment or cure for an existing condition (Keleher, 2001). Often the first point of contact in primary care in Australia is the general practitioner (physician), but this can also be the pharmacist, allied medical professional, or nurse (general practice nurse, nurse practitioners, or community nurse) (Australian Institute of Health and Welfare, 2020).

In contrast, PHC is a population level service informed by a socioecological model of health that seeks to address health inequities (unfair differences in health) and the social determinants of health that create this inequity, such as poverty, unemployment, transport issues, political structures, and geographical location (World Health Organization & United Nations Children's Fund, 2018). PHC activities cross three levels: Health promotion activities (primary level PHC), early detection, and intervention through screening (secondary level PHC) and management and rehabilitation of people with chronic disease (tertiary level PHC), using underpinning principles of individual empowerment and agency in health decisions (World Health Organization & United Nations Children's Fund, 2018). PHC can be delivered in a variety of locations including the home or community‐based settings such as local government, nongovernment not for profit agencies and other community organizations and groups, as well as in primary care settings such as general practices (Standing Council on Health, 2013).

### 1.2 The nurse's role in PHC service provision

PHC is delivered by a range of health practitioners in addition to the nurse and general practitioner, including community health workers, Aboriginal and Torres Strait Islander health practitioners, and other allied health professionals. The general practitioner is not necessarily the dominant practitioner leading and delivering these services. Indeed, nurses are the most widely distributed and accessible healthcare providers in PHC settings (World Health Organization, 2022). The nurse has a central and unique PHC role that incorporates personal care with health promotion, community empowerment, and participation. It is underpinned by the PHC principles of social justice, equity, access, and empowerment, and is informed by an understanding of social determinants of health (Australian Primary Health Care Nurses Association, 2022).

### 1.3 PHC and population health

In 2015, the United Nations developed the Sustainable Development Agenda 2030, a global blueprint to improve people's lives and protect the environment underpinned by 17 Sustainable Development Goals including “good health and well‐being” (goal 3), and “reduced inequalities” (goal 10) (United Nations, n.d.) to which Australia is invested (Australian Government, 2018). PHC services are a critical mechanism for achieving these goals (World Health Organization & United Nations Children's Fund, 2018). Countries with strong PHC systems “provide more efficient care, have lower rates of hospitalization, fewer health inequalities, and better health outcomes including lower mortality” (Standing Council on Health, 2013; p. v). The evaluation of PHC quality across service structures, processes, and outputs/outcomes is integral to this achievement (Veillard et al., 2017; World Health Organization and the United Nations Children's Fund, 2022).

### 1.4 PHC evaluation

Evaluation of quality of care is vital in improving health outcomes and assessing the effectiveness of health care services (Australian Institute of Health and Welfare, 2018). Rigorous evidence of the quality of care is needed if policy makers are to make informed decisions about health care delivery models (Laurant et al., 2018). Whilst many reliable measures exist to evaluate quality of individual medical care in acute care hospital settings (Bastemeijer et al., 2019) these are more limited in the PHC environment.

The Australian Government has recently released guidelines to resolve this discrepancy. The National Safety and Quality Primary Health Care standards, published in 2021 (Australian Commission on Safety and Quality in Health Care, 2021) aim to protect the public from harm and improve the quality of PHC delivered to patients and consumers. This document had three standards. The first “Clinical Governance Standard” outlines the responsibility of primary care and PHC practitioners to deliver safe, quality, and effective services that are continuously evaluated and improved. The standard also refers to the recognition of social determinants in this delivery of safe and effective care and outlines the importance of a service that engages in continuous quality review and improvement. Aspects of this standard cover structure, process, and outcomes of quality PHC, aligning with the Donabedian (2005) model used to organize findings in this review.

### 1.5 Purpose of the review

The purpose of this review was to explore and analyze the literature that reports on the evaluation of PHC nursing services. The review question asked, “What evaluation frameworks have been used to assess PHC nursing services and how useful are they in the Australian PHC context?”

### 1.6 The Donabedian model

**Figure 1:**
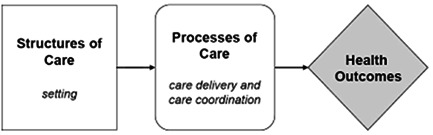
Donabedian model of quality in health care

The Donabedian model (Donabedian, 2005) continues to be a leading instrument in the evaluation of care quality in health services (Berwick & Fox, 2016). This model situates care within three areas: the structure in which care delivery is provided, the process in which engagements between patients and caregivers occur, and outcomes, which refer to the impact of care on the health status of the patient or the population (Donabedian, 2005) (Figure 1).

Structure measures (also known as input measures) consider attributes of the service/provider including staff: patient ratios, ethical approaches, times of service operation that influence service accessibility, and availability. Process measures encompass the elements of the running of the service that influence the desired outcome. For example, waiting time to be seen by a health care practitioner, staff hygiene standards, correct record keeping. Outcome measures reflect the impact of the service on patient health outcomes, and whether the desired outcomes have been met. These outcomes may include reduced mortality and morbidity, reduced hospital admissions, lifestyle and medication compliance, and improved patient experience (Donabedian, 2005). The Donabedian model is used as a framework to organize the review findings according to the area of health care assessed. The methods of evaluation used within each stage of the model are explored and examined in this review.

## 2 METHODS

The study comprised a systematic review of the literature. The protocol was informed by guidelines from Bettany‐Saltikov (2010a, 2010b).

### 2.1 Search strategy

This systematic review was initially focused on the exploration of the provision of PHC services to small, geographically isolated communities in Australia, where health services are often limited to nurse‐led PHC clinics (Muirhead & Birks, 2020). The lack of articles in the rural/remote context however meant this initial focus was abandoned. The limited availability of Australian studies also led to the inclusion of international studies in the review. Databases searched were the Joanna Briggs Institute database, MEDLINE, Proquest, and CINAHL.

Because of the confusion of terminology usage around primary care and PHC, this systematic review sought and considered articles that used the terms “primary care” or “primary health care” as potential studies relevant to the review's purpose. Those that on later examination were found not to discuss PHC (as defined in this article) were removed. The following search terms (and synonyms) were used: “primary care” OR “primary health care” OR “primary healthcare” AND nurs* AND “quality care” OR “quality eval*” OR “quality measur*” OR “quality indicator.*”

The search strategy was repeated across databases. All searches were conducted in June 2020 and again in May 2022.

### 2.2 Inclusion/exclusion criteria

**Figure 2:**
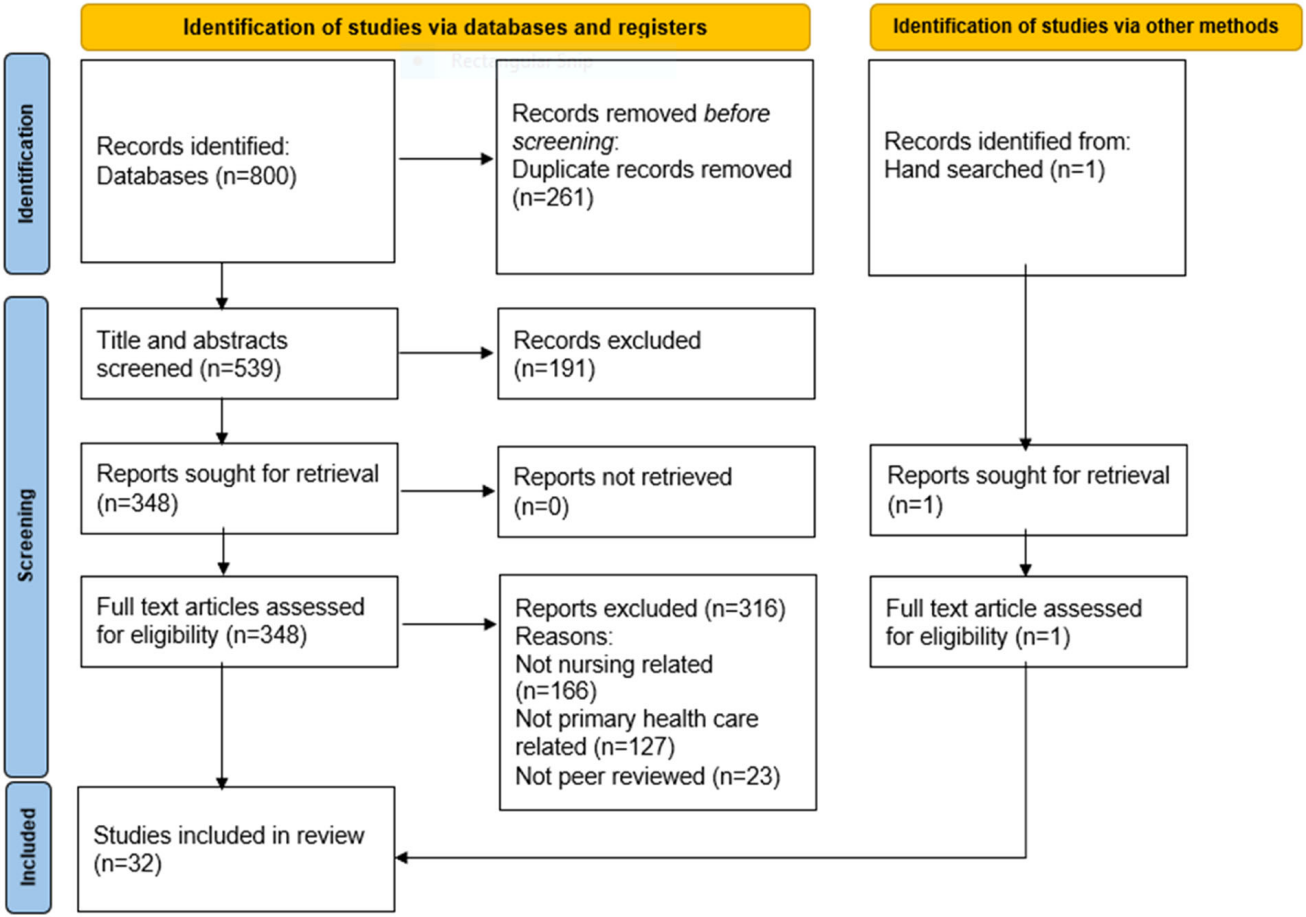
PRISMA flow diagram of literature search strategy [Color figure can be viewed at wileyonlinelibrary.com]

Papers were included if they discussed PHC services, nursing, and quality and were published between 2010 and 2022. Papers were excluded if they omitted nursing, were not in English, did not relate to quality care or if the paper was an abstract only. As the review purpose was to explore and analyze primary research that evaluated PHC services, review articles were also excluded. Three authors reviewed the articles according to the inclusion criteria and any disagreements were resolved by the entire authorship panel (Figure 2).

### 2.3 Quality appraisal

The Mixed Method Appraisal Tool (MMAT) version 2011 (Pluye et al., 2011) was used to assess the quality of research studies included in the review. Two authors applied the MMAT tool to the selected articles to ensure quality and consistency of assessment (A. G. and M. D.). The tool assesses both qualitative and quantitative research and scores each from 20% to 100% depending on the number of criteria met. These ratings were converted to a star ranking of 1 to 5 stars for ease of use (Table A1, Appendix).

The initial search from four databases resulted in 800 references. After duplicates were removed, 547 titles were assessed for eligibility. Papers meeting the inclusion criteria are presented in Appendix A as a data extraction table (Table A1, Appendix).

## 3 RESULTS

Thirty‐two studies that evaluated nursing services based in a PHC setting were included in the review. Full details are provided in Table A1 (Appendix). The date of their publication ranges from 2011 (Coddington et al., 2011; Stenner et al., 2011; Wenger et al., 2011) to 2021 (Farford et al., 2021).

### 3.1 Dates and geographical region of study

Studies were conducted in 13 countries. The majority (*n* = 11) were carried out in the United States of America (Buerhaus et al., 2018; Christiansen et al., 2016; Coddington et al., 2011; Farford et al., 2021; Jennings et al., 2016; Kinder, 2016; Kurtzman & Barnow, 2017; Mitchell et al., 2019; Poghosyan et al., 2018; Reuben et al., 2013; Wenger et al., 2011); four studies in the United Kingdom (Faulkner et al., 2016; Harris et al., 2015; Hawthorne et al., 2012; Stenner et al., 2011); three studies were Australian (Iles et al., 2014; Karnon et al., 2013; McCullough et al., 2020); three studies were undertaken in Canada (Dufour et al., 2020; Kosteniuk et al., 2017; Lawson et al., 2012); three in South Africa (Rabie et al., 2016; Rawat et al., 2018; Webb et al., 2019); and one in India (Fischer et al., 2015), Slovenia (Klemenc‐Ketis & Poplas‐Susič, 2017), The Netherlands (Vestjens et al., 2019), Sweden (Abrahamsson et al., 2015), Rwanda (Vasan et al., 2013), Argentina (Prestes et al., 2017), New Zealand (Azariah et al., 2013), and Switzerland (Gysin et al., 2020).

### 3.2 Study design and rigor

Three studies used qualitative data informed by interviews and focus groups (Fischer et al., 2015; McCullough et al., 2020; Stenner et al., 2011). Harris et al. (2015) used a mixed‐methods approach and the remaining articles used quantitative methods analyzing data from surveys, patient records, and quality audit databases. The MMAT score was applied to the research design of each article. Fifteen articles were rated as 5 star, 7 achieved 4 stars, 4 achieved 3 stars. No articles were rated below 3 stars, and therefore none were rejected for poor quality (Table A1, Appendix).

### 3.3 Study settings

The majority of the studies (*n* = 17) evaluated nursing PHC services operating within traditional physician‐led primary care community settings such as the General Practitioner clinic and other medical centers of care (Azariah et al., 2013; Buerhaus et al., 2018; Farford et al., 2021; Faulkner et al., 2016; Gysin et al., 2020; Harris et al., 2015; Hawthorne et al., 2012; Iles et al., 2014; Jennings et al., 2016; Karnon et al., 2013; Klemenc‐Ketis & Poplas‐Susič, 2017; Lawson et al., 2012; Mitchell et al., 2019; Poghosyan et al., 2018; Prestes et al., 2017; Reuben et al., 2013; Stenner et al., 2011). Fifteen studies were set in PHC settings such as remote community nursing services and urban community health centers and home visits (Abrahamsson et al., 2015; Christiansen et al., 2016; Coddington et al., 2011; Dufour et al., 2020; Fischer et al., 2015; Kosteniuk et al., 2017; Kinder, 2016; Kurtzman & Barnow, 2017; McCullough et al., 2020; Rabie et al., 2016; Rawat et al., 2018; Vasan et al., 2013; Vestjens et al., 2019; Webb et al., 2019; Wenger et al., 2011).

### 3.4 Health service type and leadership

Just 12 studies evaluated nurse‐led services, the nurses being registered community or clinic/practice nurses (Azariah et al., 2013; Coddington et al., 2011; Dufour et al., 2020; Farford et al., 2021; Fischer et al., 2015; Harris et al., 2015; Kosteniuk et al., 2017; Poghosyan et al., 2018; Vasan et al., 2013), nurse practitioners (Kinder, 2016; Stenner et al., 2011), or both (McCullough et al., 2020). Services evaluated as a whole without emphasis on provider were a primary care diabetes service (Hawthorne et al., 2012), an HIV PHC service (Rawat et al., 2018), and a PHC diabetes service (Webb et al., 2019).

More commonly, evaluated PHC services included the nurse as a member of the multidisciplinary team. Often, studies aimed to evaluate nursing care by making comparisons between the care provided by nurses and that of other health professionals, most commonly a comparison between nurse practitioners and physicians (Buerhaus et al., 2018; Christiansen et al., 2016; Gysin et al., 2020; Iles et al., 2014; Jennings et al., 2016; Karnon et al., 2013; Klemenc‐Ketis & Poplas‐Susič, 2017; Kurtzman & Barnow, 2017; Lawson et al., 2012; Mitchell et al., 2019; Prestes et al., 2017; Reuben et al., 2013; Vestjens et al., 2019; Wenger et al., 2011).

### 3.5 Structure, process, and outcome evaluation

Table 1 arranges the studies according to the Donabedian model (2005). Eleven of the 32 studies focused on an evaluation of service structure. Examples of structure evaluation include the availability of equipment in setting needed for quality diabetic care in community (Webb et al., 2019) and the geographical location of an HIV PHC service (Rabie et al., 2016).

**Table 1:** Summary of articles by Donabedian model (first author)

Donabedian model	Quality measurement via quality indicators	Quality measurement via participant experiences, perceptions
Structure	**Buerhaus** **Klemenec‐Ketis** **Reuben** **Lawson** Webb **Wenger**	**Fischer** **Harris** Kosteniuk Poghosyan Rabie
Process	Christiansen Dufour **Mitchell**	**Fischer** **Harris** Hawthorne McCullough
Outcomes	Azariah **Buerhaus** Coddington Dufour Farford Faulkner Gysin **Harris** Iles Jennings Kinder Karnon **Klemenec‐Ketis** Kurtzman **Lawson** Prestes **Reuben** Vasan **Wenger**	Abrahamsson Faulkner Kinder McCullough **Mitchell** Rawat Stenner Vestjens

Just 7 studies considered process. Examples included continuity of care in community health centers (Christiansen et al., 2016) and the mentoring process and quality of care (Fischer et al., 2015). Most commonly, the outcome of the service was evaluated (24 studies). Patient health outcomes were commonly used to measure service outcomes (see “quality indicators”).

Nine studies examined more than one aspect of the service (bold in Table 1) (Buerhaus et al., 2018; Fischer et al., 2015; Harris et al., 2015; Klemenc‐Ketis & Poplas‐Susič, 2017; Lawson et al., 2012; McCullough et al., 2020; Mitchell et al., 2019; Reuben et al., 2013; Wenger et al., 2011). Mitchell et al. (2019) for example, evaluated a primary care model in Minnesota USA, which considered staff quality of life and safety culture within the team (process), and patient experience, cancer screening targets, and care costs (outcome). Just 1 study (Harris et al., 2015) measured all three aspects of their primary care walking intervention service. This nurse‐led service was evaluated across structure (nurse and patient perceptions of equipment used), process (nurse perception of time and quality of consultations, including holistic approach), and outcomes (patient health outcomes including increased exercise duration and intensity, changes in obesity, and blood pressure).

### 3.6 Quality measures

Measurement of quality in services were carried out across two broad approaches: objective quality indicators and perceptions and experiences of services (Table 1). Just 3 studies evaluated their service using both approaches (Faulkner et al., 2016; Harris et al., 2015; Mitchell et al., 2019).

#### 3.6.1 Objective quality indicators and measurement tools

Indicators chosen to assess service quality varied. For example, Buerhaus et al. (2018) considered admission‐readmission rates, and appropriate use of clinical investigations in chronic disease management, whereas Mitchell et al. (2019) focused on affordability of care and the care‐team's work life balance with family, pediatric, and community medicine patients. Quality service decisions were also evaluated; these included appropriateness of diagnostic tests ordered and medications prescribed and diagnosis accuracy (Gysin et al., 2020; Lawson et al., 2012; Vasan et al., 2013).

Commonly, researchers measured patient health outcomes to assess service quality. These included screening rates (Azariah et al., 2013; Buerhaus et al., 2018; Christiansen et al., 2016; Gysin et al., 2020; Jennings et al., 2016; Prestes et al., 2017; Reuben et al., 2013), diabetes control (Christiansen et al., 2016; Prestes et al., 2017), obesity levels (Lawson et al., 2012) and maternal postpartum morbidity and mortality (Fischer et al., 2015).

The tools used as quality indicator measures also varied. Many studies adhered to clinical guidelines (Buerhaus et al., 2018; Christiansen et al., 2016; Coddington et. al., 2011; Dufour et al., 2020; Klemenc‐Ketis & Poplas‐Susič, 2017; Lawson et al., 2012; Poghosyan et al., 2018; Prestes et al., 2017; Reuben et al., 2013; Wenger et al., 2011). These guidelines were often devised from medical legislation and policy relevant to an individual country, such as Lawson et al. (2012) who used Nova Scotia's national and provincial clinical care indicators measuring clinical outcomes (lipids etc.), and Coddington et. al. (2011) who used national quality benchmark indicators to assess a pediatric nurse‐led clinic in Indiana. Others used internationally accepted medical tools to measure care outcomes for older adults, such as the Assessing Care of Vulnerable Elders (ACOVE) measure (Jennings et al., 2016; Reuben et al., 2013; Wenger et al., 2011).

#### 3.6.2 Service perspectives and experiences

Thirteen studies analyzed perspectives of service quality. Eight of these asked the patient/consumer (Abrahamsson et al., 2015; Faulkner et al., 2016; Harris et al., 2015; Kinder, 2016; Mitchell et al., 2019; Rawat et al., 2018; Stenner et al., 2011; Vestjens et al., 2019). Patients were asked to report on a range of service factors. For example, Kinder (2016) assessed patient satisfaction of pediatric nurse practitioner care outcomes (perceptions) as well as patient compliance to care regimen (outcome as quality indicator); Stenner et al. (2011) asked patients about the diabetic care they received from nurse prescribers in semistructured interviews.

Of the 12 studies that evaluated nurse‐led services, only four asked the perspective of the nurse (Harris et al., 2015; Kosteniuk et al., 2017; McCullough et al., 2020; Poghosyan et al., 2018).

#### 3.6.3 Evaluation tool rigor/trustworthiness

Validated tools included the ACOVE measure (Jennings et al., 2016; Reuben et al., 2013; Wenger et al., 2011) and the PES‐NWI scale to assess PHC nurse work environment (Rabie et al., 2016). Vestjens et al. (2019) used the validated Patient Assessment of Chronic Illness Care Short Version (PACIC‐S) to assess patient perceptions of quality of care with frail older people in the community.

Some researchers evaluated their services using their own tools which they validated before use. Kinder (2016) devised the PPSC‐PNP instrument to measure parental satisfaction and adherence with nurse advice, which they piloted and adjusted to strengthen reliability and validity. Kosteniuk et al. (2017) developed a scale to measure Canadian nurses' perspectives of their organization's alignment with PHC principles, reporting a Cronbach's *α* of 0.89.

The 3 qualitative studies of patients regarding nursing care used interview or focus group formats. All discussed the rigor of their study design, but none described the theory or literature used to inform question development (Fischer et al., 2015; McCullough et al., 2020; Stenner et al., 2011).

## 4 DISCUSSION

The achievement of the global Sustainable Development Goals within the Australian context, including the redress of its endemic population health inequities, is greatly reliant on quality PHC nursing services. This in turn depends on reliable and relevant evaluation systems that capture all aspects of a service (Veillard et al., 2017). To this end, the Australian Government has published The National Safety and Quality Primary Healthcare standards (Australian Commission on Safety and Quality in Health Care (ACSQHC), 2021) that clearly outline the responsibility of primary care and PHC practitioners to deliver safe, quality, and effective services that are continuously evaluated and improved. This systematic review explored the literature dedicated to the evaluation of nursing interventions in PHC, the nature of this evaluation and their potential applicability across Australian PHC contexts. Four main issues arising from this review are now discussed.

### 4.1 A need for nurse‐led service model evaluation

The review revealed a heterogenous, disjointed approach to PHC service evaluation. Despite their importance to global health and the achievement of the Sustainable Development Goals, comparatively few studies have evaluated nursing PHC services world‐wide.

Of the three Australian PHC services evaluated, just one (by McCullough et al., 2020) was a nurse‐led study. A more common approach to service evaluation, particularly in the United States of America, was the comparison of the quality of nurse practitioner and physician care in the primary care setting. While nurse practitioners are now commonplace in United States of America health service delivery, Australia appears more cautious in accepting them as health care professionals (Carter et al., 2015). Further, there were no studies that compared the quality of care between nurse practitioners and registered nurses, which limits the evaluation of differences in nursing service provision. The findings from future studies that demonstrate the efficacy of nurse practitioners, as compared to general practitioners and registered nurses, may guide workforce development and justify acceptance as autonomous service providers in Australia.

The narrow approach of these studies in terms of their focus on medical outcome measurements (such as diagnosis and prescribing costs) and the lack of focus on the unique attributes of the nurse in the PHC role makes them less helpful to the development of an understanding the efficacy and quality of nursing PHC services. The evaluation nursing attributes, including the nursing skills and qualities that improve patient outcomes—such patient health literacy support, advocacy, and empowerment are crucial.

### 4.2 Capturing the nurse perspective

The comparatively high number of studies that included the patient voice in service evaluation is to be commended, aligning well with the PHC principle of patient participation in health service development and provision (World Health Organization and the United Nations Children's Fund, 2022). The finding that nurses were less frequently asked to contribute their perspectives and experiences within the evaluation of the care they provide concurs with a previous literature review (Ryan et al., 2017). This oversight is concerning. Already a relatively disempowered health profession (Burton, 2020), this further disenfranchises the nurse as an autonomous leader and decision maker in health. It also diminishes the nurse's role as patient advocate and their capacity to apply their holistic knowledge of community to inform the development of an accessible and acceptable PHC service model.

### 4.3 A structure‐process‐outcome focus

PHC scholars argue that quality measurement of a PHC service must involve the interplay between service structure, process, and outcome (Arvidsson et al., 2019; Berwick, 1989; Berwick & Fox, 2016). Many of the studies in this review limited their PHC service evaluation to the outcome section of the Donabedian (2005) model. This focus, to the exclusion of structures and processes, appears common in PHC service evaluation literature (see Lukewich et al., 2022; Simou et al., 2015). Studies that consider patient health outcomes but not service structure and processes ignore the holistic nature of services and the association between these factors and service outcomes. The outcome of a screening service uptake is, for example, greatly influenced by the service structure (accessibility) and process (acceptability). Ignoring factors such as the cultural appropriateness, cost and setting of a screening service limits the usefulness and meaningfulness (the why?) of the outcome findings (Penchansky & Thomas, 1981).

### 4.4 Shifting away from the medical lens

Although study designs and evaluation tools were generally acceptable in terms of rigor, the lens through which many services were assessed for quality was less ideal. Many of the quality criteria used appeared to be highly medical in nature (such as diagnosis rates, diagnosis accuracy, and pain outcomes). The ANCOVE measure, for example, whilst validated and widely accepted in the acute medical setting, is an individualized disease‐centered tool with limited applicability in the PHC environment.

This disconnect with PHC principles reduces the applicability of these evaluation approaches to PHC services. One study in the review that demonstrated a contrasting approach was Kosteniuk et al. (2017) who developed a tool to measure nurses' perspectives of their organization's PHC service and its alignment with PHC principles. The evaluation was underpinned by principles of PHC including service accessibility/availability, holism, comprehensiveness, and a multidisciplinary team approach. This approach provided a way of assessing many aspects of structure and process quality of PHC services, although not outcomes, using PHC concepts that are transferable across national and cultural divides.

The setting of the majority of the reviewed PHC service evaluations also reflected the medical model, being set in the primary care environment, in particular in general practice (community physician setting). Often based on private business models, general practitioner‐led primary care services tend to lack service integration, carry out insufficient levels of prevention work and may lack accessibility to poorer people, reducing their potential for equity (Swerissen et al., 2018). As such, by their very nature, they are unable to provide a base for quality PHC service delivery.

### 4.5 Implications for practice

Without an accepted PHC nursing service evaluation approach, the quality of equity focused service provision cannot be ascertained. This has significant implications for the effectiveness of nursing services that aim to redress local health disparities and the wider achievement of the global Sustainable Development Goals.

An outcome of this review is the presentation of a range of methods for evaluation that may be replicated. Researchers and clinicians may consult the data extraction table for inspiration in conducting their own data collection and analysis of PHC programs and assessment of nursing services. Further, changes to workforce and service provision as a result of the Australian Government's (2021, 2022) recently released PHC 10‐year plan, may be monitored and evaluated using methods presented in this review. However, of note, is the limited mention of the roles of nurses within this plan.

## 5 LIMITATIONS

This review was limited to peer‐reviewed academic literature and as such did not consider studies found in the gray literature. Expert opinion pieces or articles published in a language other than English were also not considered. A further limitation of this review is variation in the way primary care and PHC may be interpreted. While the initial inclusion of the search term “primary care” ensured “mistermed” PHC studies were not excluded for review, it is reasonable to assume that some PHC articles were missed in cases where other terminology has been used to describe these services.

## 6 CONCLUSIONS AND RECOMMENDATIONS

The Australian Government's National Safety and Quality Primary Healthcare Standards (ACSQHC, 2021) has recently called for PHC providers to evaluate their services for quality and governance standards.

This review has demonstrated that, in contrast with the acute medical setting, there is a paucity of approaches designed to measure this, especially those led by the nurse. An examination of the existing approaches reveals many researchers have applied a medical model approach, including medical indicators and a limited focus on patient disease outcomes to understand the quality of PHC nursing services. Few have approached their evaluation through the lens of PHC principles, or have involved the nurse experience, and many lack a holistic structure‐process‐outcome approach to their evaluation. The usefulness of these models as tools to evaluate PHC services tasked with the achievement of national and international population health goals is therefore questionable.

A coordinated approach is required, based on PHC principles to understand the unique contribution of the nurse to PHC population health in Australia. This requires a mind‐shift in which nurses are consulted and trusted to lead services and the process of service evaluation that is based on a holistic approach to this evaluation based on a framework of PHC principles.

## AUTHOR CONTRIBUTIONS

**Kylie McCullough, Lesley Andrew, Angela Genoni, Melissa Dunham, Lisa Whitehead, Davina Porock**: Substantial contributions to the conception or design of the work; or the acquisition, analysis, or interpretation of data for the work. Drafting the work or revising it critically for important intellectual content. Final approval of the version to be published. Agreement to be accountable for all aspects of the work in ensuring that questions related to the accuracy or integrity of any part of the work are appropriately investigated and resolved.

## CONFLICT OF INTEREST

The authors declare no conflict of interest.

### 1 PEER REVIEW

The peer review history for this article is available at https://publons.com/publon/10.1002/nur.22291.

## Data Availability

This is a systematic literature review. Data are drawn from published research articles.

## APPENDIX A

**Table A1:** Data extraction table

Donabedian model	First author, date of publication, country	Care providers/service under evaluation	Research aims	Methods	Evaluation tools/data. Key findings	Nurse led?	MMAT score
Outcomes	Abrahamsson (2015) Sweden	PHC service.	To determine factors associated with patient recommendations to a PHC.	Cross‐sectional study, response to binary question: Would you recommend the visited primary health care center?	Swedish national patient survey. Recommendations based encounter responsiveness was strongly associated with patient tendency to recommend the primary healthcare center. The profession (nurse or physician) involved in the treatment encounter made no difference to the predictive significance.	N	****
Outcomes	Azariah (2013) New Zealand	Primary care registered nurse Chlamydia screening service.	To pilot a chlamydia screening nurse‐led service.	Laboratory testing data were analyzed over the 4‐month pilot project.	Statistically significant increase in opportunistic chlamydia screening in practice population during nurse‐led pilot.	Y	****
Structure and outcomes	Buerhaus (2018) USA	Primary care physicians and nurse practitioner.	To examine differences in quality of care provided by primary care physicians or nurse practitioners.	2012/3, retrospective cohort study assessing 16 claims‐based quality measures.	Quality of care was compared between NPs and PCPs. Data were USA Medicare and Medicaid billing files and enrollment records. Patients assigned to NPs had lower hospital admissions, readmissions, less inappropriate use of ED, and imaging for low back pain. Those assigned to PCPs had better chronic disease management care.	N	*****
Process and outcomes	Christiansen (2016) USA	Community registered nurses and primary care practitioner.	To determine if assigning patients to a designated primary care provider improves quality of care.	Quality, continuity and efficiency measures collected at baseline, 6 and 12 months.	Defined by continuity and efficiency of patient care. Measured via five health indicators. Completion of cervical and colorectal cancer screenings, diabetes control indicated by blood pressure, LDL, and HbA1c. Quality indicators improved by 9% via assignment to dedicated practitioner (not distinguished between nurses and physicians).	N	*****
Outcomes	Coddington (2011) USA	Nurse‐managed community pediatric clinic.	To evaluate quality care in a nurse managed center.	Nonexperimental design, retrospective data collection from medical chart data.	Defined by HEDIS quality indicators used in the study Immunization status, appropriate treatment for respiratory infections, access to primary care providers, well‐care checks. HEDIS quality indicators were all met or exceeded by nursing staff.	Y	*****
Process and outcomes	Dufour (2020) Canada	Primary care nurses.	To measure and validate performance indicators that are sensitive to primary care nursing, using wound care as an indicator.	Longitudinal study. Retrieval of electronic patient information for those receiving wound care for longer than 7 days.	Eight nursing sensitive indicators were examined. Five related to process and three to outcomes. Process indicators: Nursing follow‐up, continuity, education, initial assessment, specialized nurse. Outcome indicators: frequency, duration, and intensity. Correlations between process and outcome indicators were examined. Associations between nursing follow‐up, continuity indicators, and outcome indicators were significant.	Y	*****
Outcomes	Farford (2021) USA	Primary care nurses.	To evaluate registered nurse service impact on preventative service outcomes in a family medical clinic.	Retrospective patient chart review over 1 year.	Nurse‐led service was an effective way to support patient preventative health needs.	Y	****
Outcomes	Faulkner (2016) UK	Practice nurses and nursing auxiliary working in primary care.	To measure the difference in care delivered by both healthcare worker groups in smoking cessation support services.	Data from iQuit randomized controlled trial. Measurements were abstinence, patient satisfaction, and consultation time.	No statistically significant difference between the healthcare workers with either criteria.	N	****
Structure and process	Fischer (2015) India	PHC clinic nurses.	To describe the experience of implementing quality improvement intervention in maternal and newborn care through mentoring in PHCs.	Mentoring program involving nurse mentors across 385 PHC clinics in rural India.	Quality care defined by reduced labor, delivery, and postpartum maternal morbidity and mortality. Observations, focus groups, and interview data were collected. Five field visits were conducted. Observation checklists were used but not provided insufficient detail in supplementary information. Mentoring delivered an increase in PHC nurse ability to provide care according to guidelines and to handle complications.	Y	****
Outcomes	Gysin (2020) Switzerland	Nurse practitioners and primary care physicians in a single primary care practice.	To gain insight into patient characteristics and services delivered in nurse practitioner consultations compared to physician consultations.	Collation of retrospective observational data for nurse practitioner delivered consultations compared to those delivered by a physician.	Case‐study used retrospective observational data from medical records. Patient characteristics, services delivered, chronic conditions medicated were obtained and compared between nurse and physician. Nurse practitioners measured vital signs and anthropometric data more frequently, and ordered lab tests more often than the physician, independent of patient characteristics. By contrast, medications were prescribed or changed less frequently by nurse practitioners. Nurse practitioner care may have advantages for providing care to growing number of patients with multimorbid conditions.	N	***
Structure, process, and outcomes	Harris (2015) UK	Primary care nurse delivered walking intervention service for older adults.	To evaluate if the service increased step count and moderate to vigorous walking activity among service participants.	Cluster Randomized controlled trial. Plus semistructured interviews with participants and nurses. Participant data pre and postintervention including obesity, blood pressure, steps walked. Participant and nurse perspectives on service.	Improvements in step count and exercise intensity only in intervention group. Positive findings on patient and nurse feedback of service. Good level of acceptability and uptake of service.	Y	*****
Process (outcome secondary)	Hawthorne (2012) UK	Primary care diabetes service.	To investigate care of patients with type 2 diabetes from patient and healthcare professional and patient perspective.	Patients with type 2 diabetes, physicians and nurses sent questionnaire re: management of type 2 diabetes.	Survey on receival of specific elements of care, confidence in condition management, and preventive care for comorbid conditions. Derived from UK NHS Healthcare Commission questionnaire. Clinician survey included questions on routine behaviors such as weight management, referrals to specialists for exercise and diet. Clinicians reported giving advice more often than patients reported receiving it. Correlations between clinician and patient report were low. A wide range of patient reported a lack of confidence with managing their condition (3%−39%).	N	***
Outcomes	Iles (2014) Australia	Practice nurse and physician.	Determine economic feasibility of practice nurse‐led model of chronic disease management.	12‐month longitudinal observation study. 12 months of patient record data and Medicare charges analyzed.	Costs associated with nurse‐led care were higher than physician led care, however these costs were manageable within the practice budget.	N	****
Outcomes	Jennings (2016) USA	Nurse practitioners and primary care physicians.	To assess the quality of dementia and Alzheimer's care provided by nurse practitioners in codoctor‐nurse designed delivery model	Review of medical charts for the first (*n* = 797) patients enrolled the Alzheimer/Dementia Care program.	Extraction of care provided from medical records using ACOVE‐Quality indicators. Comprehensive dementia care comanagement with NP resulted in high quality of care for dementia in the areas of assessment, screening, and counseling.	N	*****
Outcomes	Karnon (2013) Australia	Practice nurses and primary care physicians.	Comparison of cost of obesity management primary care service.	Longitudinal observational study. 12 months of medical record data.	High levels of practice nurse involvement increased service costs but potentially improved patient quality of life measures.	N	****
Outcomes	Kinder (2016) USA	Pediatric nurse practitioner.	Explore parent perception of satisfaction of care from pediatric nurse practitioners (PNPs).	Descriptive correlational research design.	Parent perception of Satisfaction with Care from Pediatric Nurse Practitioners tool was used to assess four components of satisfaction and overall satisfaction: communication, clinical competence, caring behavior, and decisional control. Clinical competence had the strongest positive relationship with parental intent. Clinical competence is important in the PNP role.	Y	*****
Structure and outcomes	Klemenec‐Ketis (2017) Slovenia	Physicians, nurse practitioners and practice nurses.	To determine the associations between selected quality indicators and characteristics of the team members, to promote quality improvement.	Cross‐sectional study. Data gathered from automatic electronic reports on quality indicators.	Measured via examination of quality indicators relating to chronic disease. Foot examination in patients with diabetes once per year, and mean value of BP in chronic patients (140/90 mmHg or lower). Also determined HbA1C in diabetic patients. Quality indicators were associated with training. NPs require continuous professional development to effectively manage patients with chronic disease.	N	***
Structure	Kosteniuk (2017) Canada	Rural and remote registered nurses.	To create an instrument to reflect essential components of PHC.	Nurse survey. Consultations with expert nurse team to identify essential components of rural and remote care.	A 28 item primary health care engagement scale was developed and validated. The scale measures accessibility/availability, community participation, and intersectoral teamwork.	Y	****
Outcomes	Kurtzman (2017) USA	Nurse practitioners, primary care physicians, and nurse assistants.	To compare quality of care and practice patterns between nurse practitioners, physician assistants, and physicians.	Retrospective study using 5 years data (2006−2010) from community healthcare centers.	Measured via 9 patient level outcomes, 3 quality indicators, 4 service utilization measures, and 2 referral pattern measures. In 7 of 9 outcomes, no significant differences was detected between nurse practitioners and physician care. Nurse practitioners provided more smoking cessation counseling.	N	*****
Structure and outcomes	Lawson (2012) Canada	Physicians and nurse practitioner in one rural family practice.	To evaluate the effect of an enhanced collaborative PHC model, including physicians and NP team members.	Observational retrospective, patient chart‐audit, pre and postimplementation of collaborative PHC model.	Chronic condition quality indicators associated with specific age and sex‐by‐age subgroups (*n* = 15) focused on preventive measures. Improved chronic disease management for chronic disease where a NP is part of the collaborative team.	N	*****
Structure process and outcomes	McCullough (2020) Australia	Community and remote community nurses, nurse practitioners, nurse academics.	Describe and explain the actions and interactions involved in the delivery of quality PHC in remote communities.	Grounded theory.	An interview guide with broad open‐ended questions explored the participants' experiences of nursing in a remote setting. Core issue was the inability to provide primary healthcare. Four issues were related to this: understanding the social world of the remote community, availability of resources, clinical knowledge and skill, shared understanding, and support.	Y	*****
Structure process and outcomes	Mitchell (2019) USA	Primary health center practitioners including GPs, registered nurses, allied health.	Research aims in four areas: Improving population health, improving patient experience, reducing costs, and supporting care team's work life.	Implementation of a team‐based care model and evaluation of quality improvement data.	Quality metrics assessed: population health, operational process measures, patient experience and satisfaction, affordability of care and the care team's work life. Team based models well accepted by patients and team members, supported by quality care provided.	N	*** (quality metrics methodology unclear)
Structure	Poghosyan (2018) USA	Primary care nurses in primary care practice.	To investigate the relationship between nurse practitioner practice environments and quality of care for chronic diseases.	Nurses surveyed to assess organizational environment, correlated with patient care data for three chronic diseases, asthma, diabetes, and CVD.	Nurse practitioner primary care organizational climate questionnaire, with four subscales: relationships with physicians, independent practice and support, professional visibility and, relationships with administration. Increase in organizational level nurse practitioner administration relations subscale associated with an almost double odds of receiving asthma medication management. Increase in the independent practice and support subscale associated with 60% increase in screening for CVD.	Y	****
Outcomes	Prestes (2017) Argentina	Primary care practitioners and registered nurses.	To determine follow‐up effect of quality of care provided to diabetic patients after implementation of system changes.	Intervention versus control study.	Adherence to treatment guidelines, defined by quality indicators. The intervention included a diabetes training course for physicians and course for nurses. Patients were followed up every 3 months. Qualidiab data system was used for evaluation. No significant differences between groups, with both groups not performing sufficient quality care requirements.	N	*****
Structure	Rabie (2016) South Africa	Professional nurses.	To describe the demographic of community healthcare nurses and the status of the practice environment, using a validated questionnaire.	Cross sectional study of professional nurses. Surveyed using practice environment scale of the nursing work index (PES‐NWI).	Study identified and described the status of practice environments in South Africa, using the PES‐NWI. Subscales for lack of staffing and participation in primary healthcare staffing scored low, however, other subscales scored highly, including quality of care.	N	****
Outcomes	Rawat (2018) South Africa	HIV primary healthcare service.	To determine if patients and caregivers reported changes in quality of care and satisfaction with staff during a 10‐month period.	Survey of patients and caregivers across four clinics and two time points.	Survey designed and validated for the province of administration, used 5‐point Likert scales for 14 components of quality care and 8 components of staff satisfaction. Quality of care scores were higher for ages 36−45 versus 18−25 years, and lower for those attending clinics for more than 10 years versus 6−12 months. Satisfaction with staff scores were lower for child health attendees and higher for tuberculosis attendees compared to chronic disease care attendees.	N	****
Structure and outcome	Reuben (2013) USA	Primary care physicians and nurse practitioners.	Assessing quality of care of older patients through physician/nurse comanagement.	Patients who received comanagement compared to physician alone.	Quality of care assessed from medical records review using ACOVE‐3 Qis. Comanagement was significantly associated with receiving recommended care. C‐management was associated with better quality care for falls, urinary incontinence and dementia.	N	*****
Outcomes	Stenner (2011) UK	Nurse prescribers in primary care setting.	Explore views of patients with diabetes about consultations with nurse prescribers and the impact on medication management.	Semistructured interviews and thematic analysis of patients with diabetes in 2009.	Thematic analysis revealed three main themes. Nurse consultation style, benefits of the nurse prescriber consultation, and views on involvement and decision making. “Continuity of relationship, flexibility over consultation length, nurses' interpersonal skills, and specialist diabetes knowledge were considered crucial to good quality care.”	Y	*****
Outcomes	Vasan (2013) Rwanda	Registered nurses in out‐patient community clinics.	To describe the routine quality of care for adults and adolescents in rural Rwanda to enable identification of needs for improvement.	Nurse delivered care to patients were observed during 2011 for patients aged 13+ and assessed for meeting care delivery guidelines.	Quality defined as receipt of complete medical service including triage, screening, diagnosis, and treatment. Measured via mentors trained in WHO Integrated Management of Adolescent and Adult Illness protocol using standard checklists. Of 470 observed consultations just 1.5% screened and triaged for emergency conditions. Fewer than 10% of patients were routinely screened for chronic conditions including HIV, tuberculosis, anemia, or malnutrition. Nurses correctly diagnosed 50.1% of patient complaints and determined the correct treatment 44.9% of the time. Correct diagnosis and treatment varied significantly across health centers.	Y	****
Outcomes	Vestjens (2019) Netherlands	General practitioners and practice nurses.	To examine whether frail community‐dwelling older persons perspective on quality of primary care associated with patient‐professional interactions.	Quasi‐experimental design assessing quality of care and patient, nurse, and doctor interactions for frail, community dwelling older adults at baseline and 12 months.	Patient perceptions of quality measured via Patient Assessment of Chronic Illness Care Short version (PACIC‐S). Frail community‐dwelling older persons' perspectives on quality of primary care were associated with their perceptions of interactions with the general practitioner and practice nurse in both groups. There were no significant differences in overall perceived quality of care between groups and at follow‐up.	N	*****
Outcomes	Webb (2019) South Africa	PHC clinic diabetes service.	To assess capacity of primary care clinics to provide quality diabetes care.	Audit tool developed assessing competence standards. Interviews with managers, nurses, or pharmacists.	Assessed using self‐developed audit tool, based on national and clinical guidelines. The results indicated care may be compromised by the poor availability of guidelines, educational material, and the absence of monofilaments.	N	*****
Structure and outcome	Wenger (2011) USA	Community registered nurses and primary care practitioner.	To evaluate the quality of care of the nurse care management model in the community setting.	Electronic record retrieval over a 13‐month period.	Evaluation and comparison of care received by both clinician and NP by comparison of quality indicators. Defined by 107 ACOVE quality indicators. Addition of nurse practitioner to special needs plans, recommended care adherence increased to 69% (compared to 53% for GP only).	N	*****

*Note*: Asterisk in last column indicate that the scale is from 1 to 5 *. Only articles with 3 and above * were included in the study.

Abbreviations: ACOVE, assessing care of vulnerable elders; MMAT, mixed method appraisal tool; PHC, primary health care.
